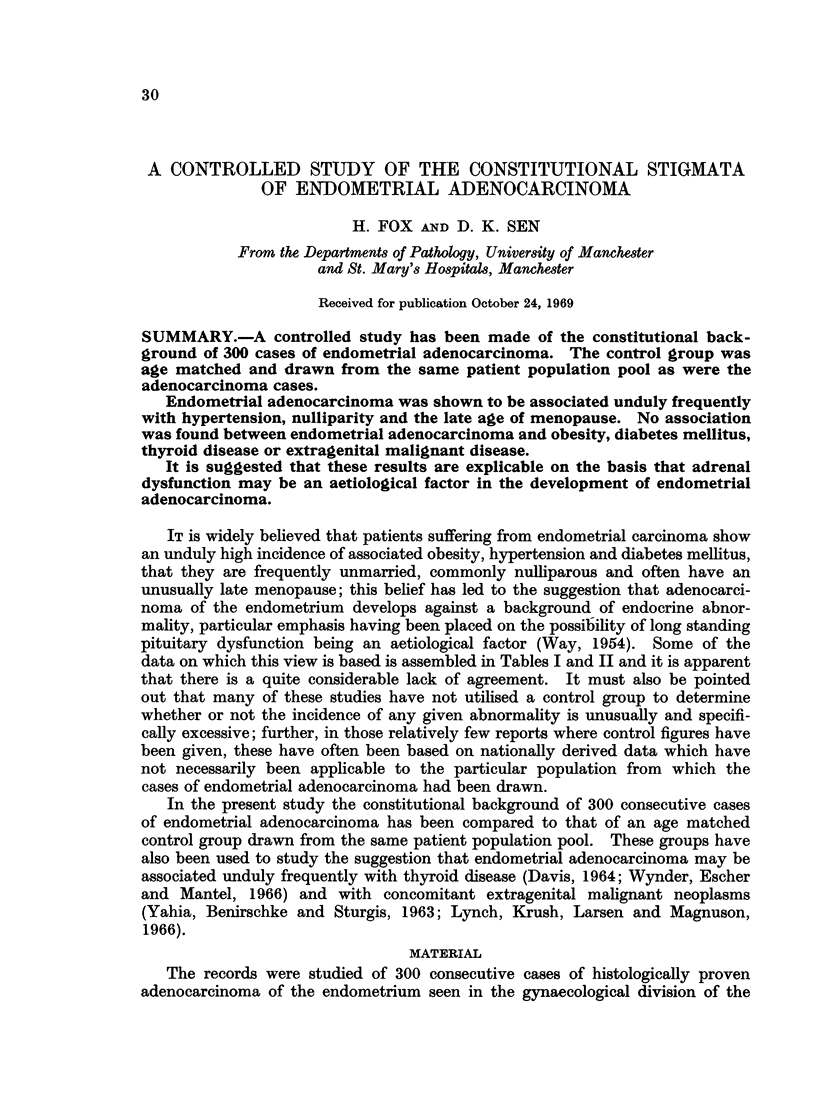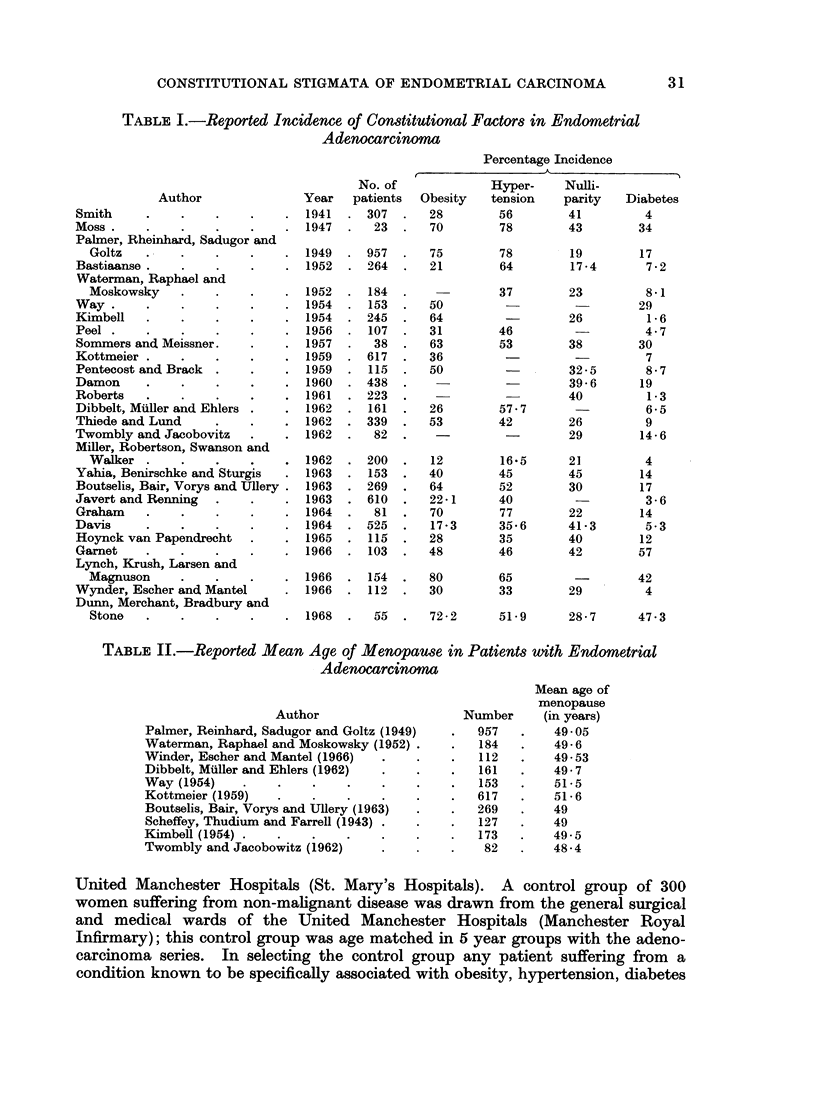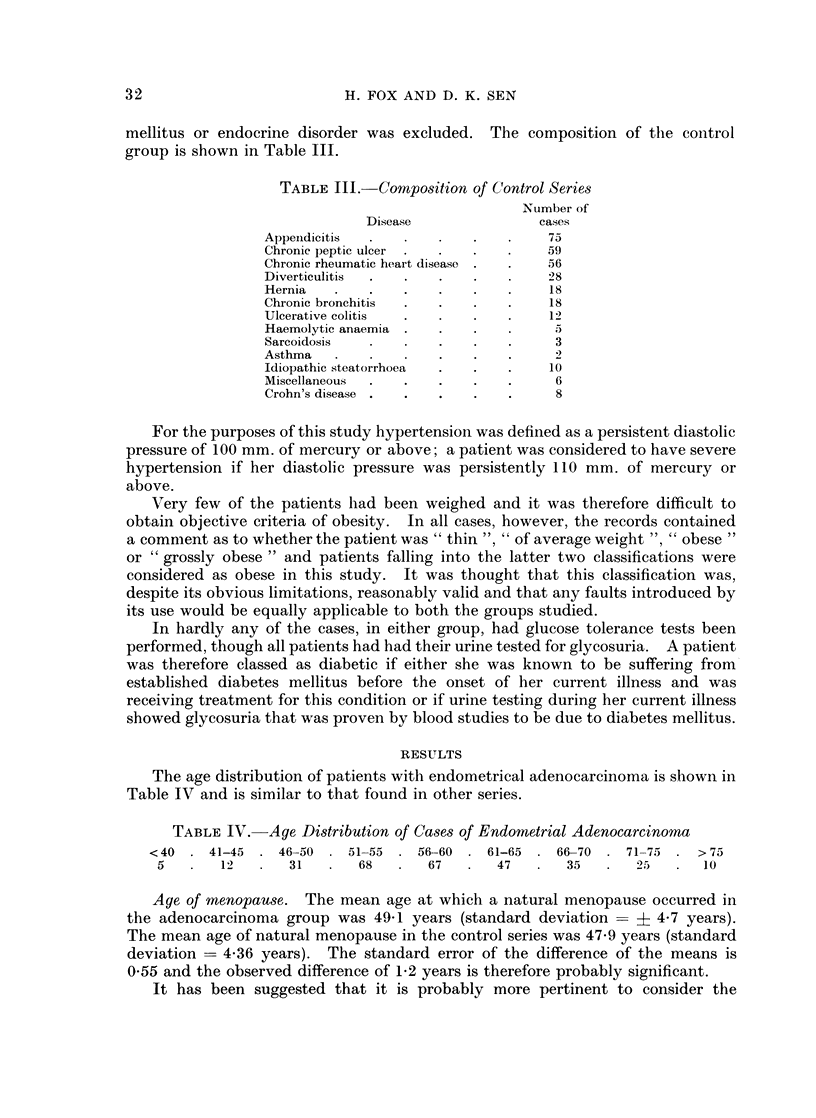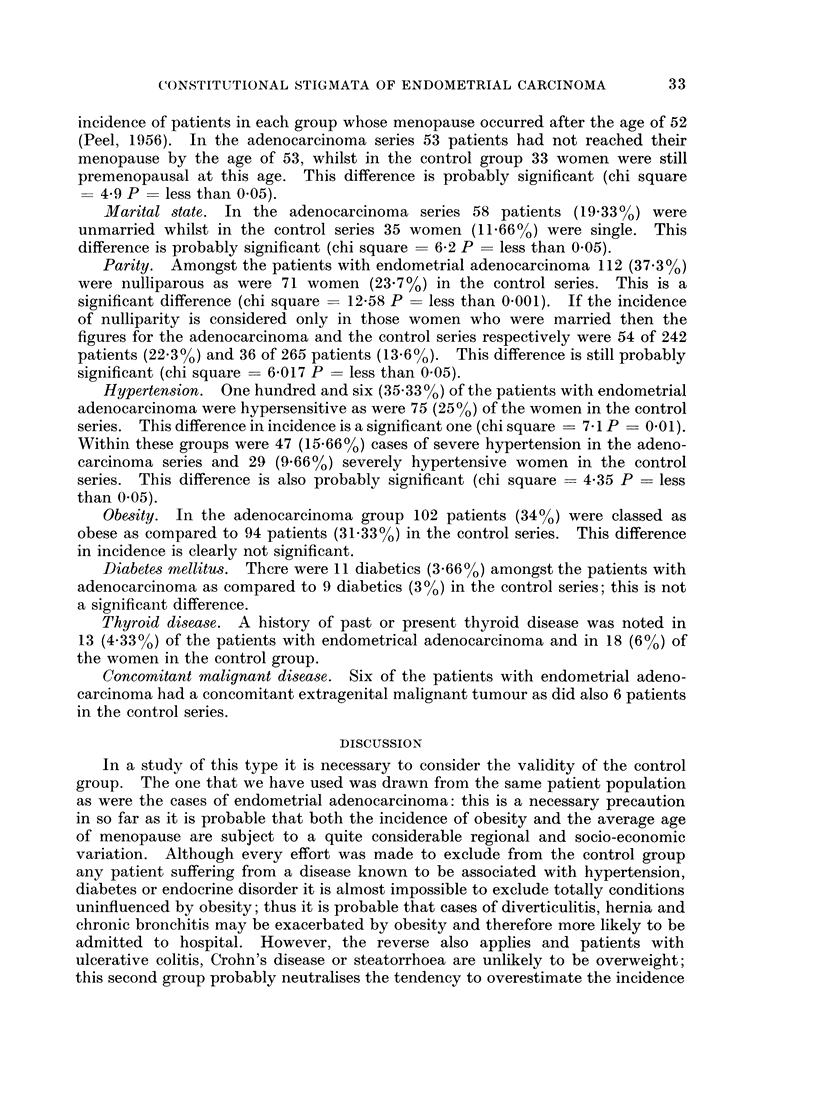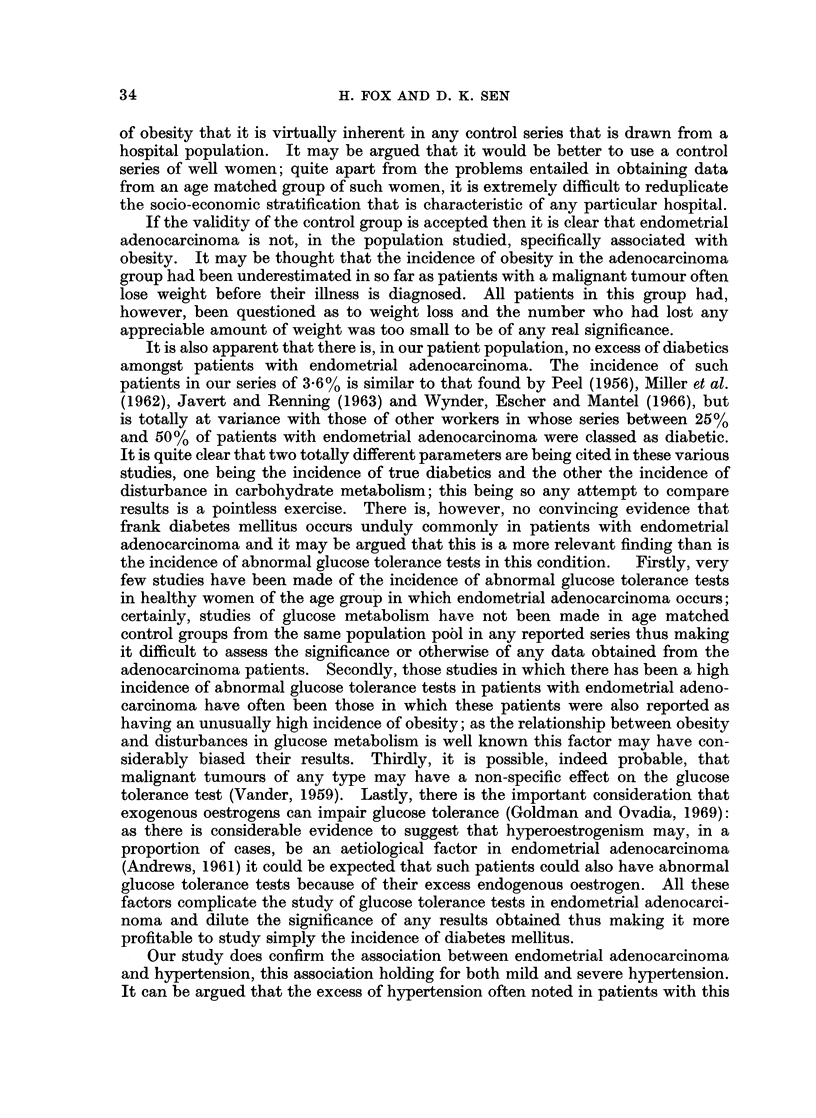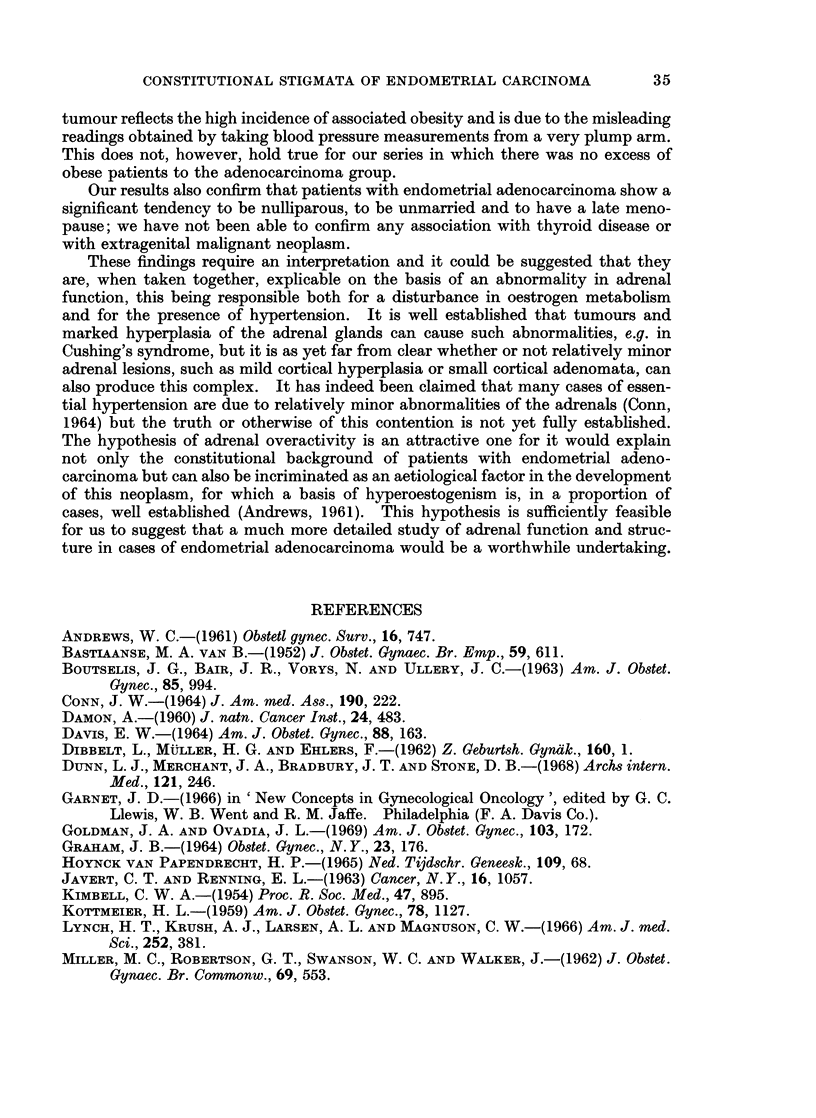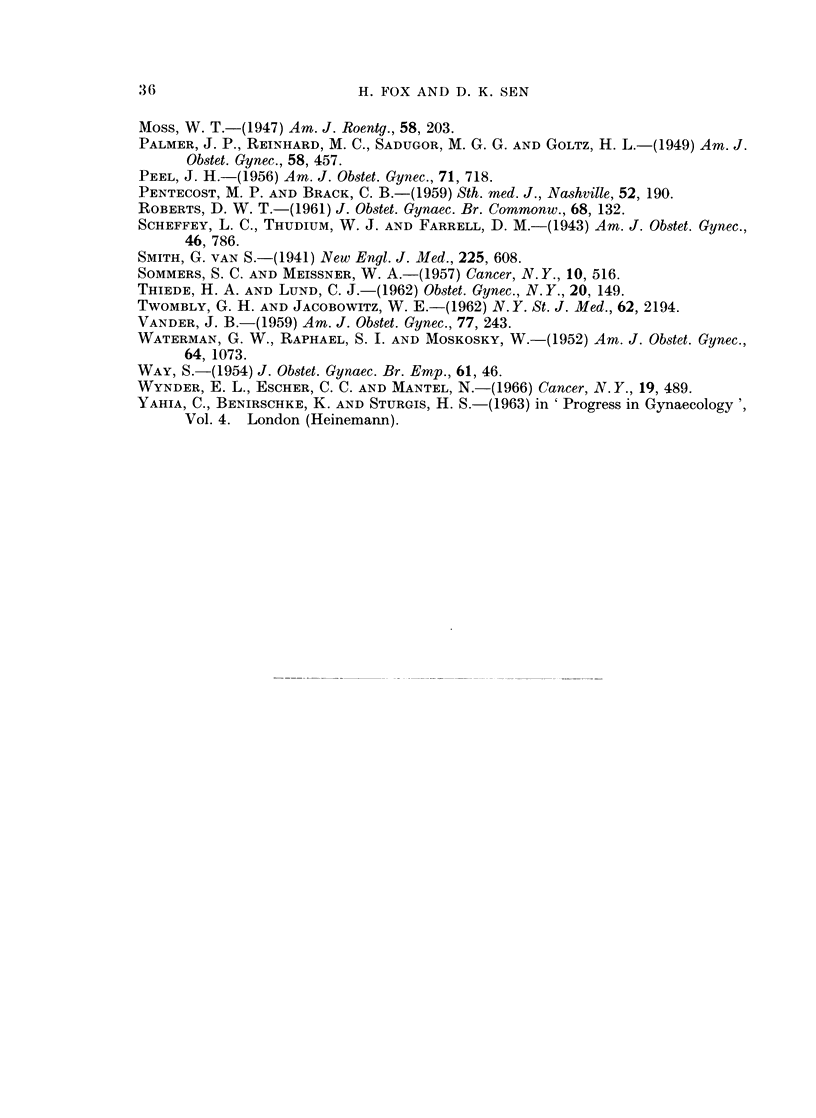# A Controlled Study of the Constitutional Stigmata of Endometrial Adenocarcinoma

**DOI:** 10.1038/bjc.1970.5

**Published:** 1970-03

**Authors:** H. Fox, D. K. Sen

## Abstract

A controlled study has been made of the constitutional background of 300 cases of endometrial adenocarcinoma. The control group was age matched and drawn from the same patient population pool as were the adenocarcinoma cases.

Endometrial adenocarcinoma was shown to be associated unduly frequently with hypertension, nulliparity and the late age of menopause. No association was found between endometrial adenocarcinoma and obesity, diabetes mellitus, thyroid disease or extragenital malignant disease.

It is suggested that these results are explicable on the basis that adrenal dysfunction may be an aetiological factor in the development of endometrial adenocarcinoma.


### A CONTROLLED STUDY OF THE CONSTITUTIONAL STIGMATA OF ENDOMETRIAL ADENOCARCINOMA

H. FOX AND D. K. SEN

*From the Departments of Pathology, University of Manchester and St. Mary's Hospitals, Manchester*

Received for publication October 24, 1969

**SUMMARY.—A controlled study has been made of the constitutional background of 300 cases of endometrial adenocarcinoma. The control group was age matched and drawn from the same patient population pool as were the adenocarcinoma cases.**

**Endometrial adenocarcinoma was shown to be associated unduly frequently with hypertension, nulliparity and the late age of menopause. No association was found between endometrial adenocarcinoma and obesity, diabetes mellitus, thyroid disease or extragenital malignant disease.**

**It is suggested that these results are explicable on the basis that adrenal dysfunction may be an aetiological factor in the development of endometrial adenocarcinoma.**

IT is widely believed that patients suffering from endometrial carcinoma show an unduly high incidence of associated obesity, hypertension and diabetes mellitus, that they are frequently unmarried, commonly nulliparous and often have an unusually late menopause; this belief has led to the suggestion that adenocarcinoma of the endometrium develops against a background of endocrine abnormality, particular emphasis having been placed on the possibility of long standing pituitary dysfunction being an aetiological factor (Way, 1954). Some of the data on which this view is based is assembled in Tables I and II and it is apparent that there is a quite considerable lack of agreement. It must also be pointed out that many of these studies have not utilised a control group to determine whether or not the incidence of any given abnormality is unusually and specifically excessive; further, in those relatively few reports where control figures have been given, these have often been based on nationally derived data which have not necessarily been applicable to the particular population from which the cases of endometrial adenocarcinoma had been drawn.

In the present study the constitutional background of 300 consecutive cases of endometrial adenocarcinoma has been compared to that of an age matched control group drawn from the same patient population pool. These groups have also been used to study the suggestion that endometrial adenocarcinoma may be associated unduly frequently with thyroid disease (Davis, 1964; Wynder, Escher and Mantel, 1966) and with concomitant extragenital malignant neoplasms (Yahia, Benirschke and Sturgis, 1963; Lynch, Krush, Larsen and Magnuson, 1966).

### MATERIAL

The records were studied of 300 consecutive cases of histologically proven adenocarcinoma of the endometrium seen in the gynaecological division of the

TABLE I.—*Reported Incidence of Constitutional Factors in Endometrial Adenocarcinoma*

| | | | Percentage Incidence | | | |
|---|---|---|---|---|---|---|
| Author | Year | No. of patients | Obesity | Hyper-tension | Nulli-parity | Diabetes |
| Smith | 1941 | 307 | 28 | 56 | 41 | 4 |
| Moss | 1947 | 23 | 70 | 78 | 43 | 34 |
| Palmer, Rheinhard, Sadugor and Goltz | 1949 | 957 | 75 | 78 | 19 | 17 |
| Bastiaanse | 1952 | 264 | 21 | 64 | 17·4 | 7·2 |
| Waterman, Raphael and Moskowsky | 1952 | 184 | — | 37 | 23 | 8·1 |
| Way | 1954 | 153 | 50 | — | — | 29 |
| Kimbell | 1954 | 245 | 64 | — | 26 | 1·6 |
| Peel | 1956 | 107 | 31 | 46 | — | 4·7 |
| Sommers and Meissner | 1957 | 38 | 63 | 53 | 38 | 30 |
| Kottmeier | 1959 | 617 | 36 | — | — | 7 |
| Pentecost and Brack | 1959 | 115 | 50 | — | 32·5 | 8·7 |
| Damon | 1960 | 438 | — | — | 39·6 | 19 |
| Roberts | 1961 | 223 | — | — | 40 | 1·3 |
| Dibbelt, Müller and Ehlers | 1962 | 161 | 26 | 57·7 | — | 6·5 |
| Thiede and Lund | 1962 | 339 | 53 | 42 | 26 | 9 |
| Twombly and Jacobovitz | 1962 | 82 | — | — | 29 | 14·6 |
| Miller, Robertson, Swanson and Walker | 1962 | 200 | 12 | 16·5 | 21 | 4 |
| Yahia, Benirschke and Sturgis | 1963 | 153 | 40 | 45 | 45 | 14 |
| Boutselis, Bair, Vorys and Ullery | 1963 | 269 | 64 | 52 | 30 | 17 |
| Javert and Renning | 1963 | 610 | 22·1 | 40 | — | 3·6 |
| Graham | 1964 | 81 | 70 | 77 | 22 | 14 |
| Davis | 1964 | 525 | 17·3 | 35·6 | 41·3 | 5·3 |
| Hoynck van Papendrecht | 1965 | 115 | 28 | 35 | 40 | 12 |
| Garnet | 1966 | 103 | 48 | 46 | 42 | 57 |
| Lynch, Krush, Larsen and Magnuson | 1966 | 154 | 80 | 65 | — | 42 |
| Wynder, Escher and Mantel | 1966 | 112 | 30 | 33 | 29 | 4 |
| Dunn, Merchant, Bradbury and Stone | 1968 | 55 | 72·2 | 51·9 | 28·7 | 47·3 |

TABLE II.—*Reported Mean Age of Menopause in Patients with Endometrial Adenocarcinoma*

| Author | Number | Mean age of menopause (in years) |
|---|---|---|
| Palmer, Reinhard, Sadugor and Goltz (1949) | 957 | 49·05 |
| Waterman, Raphael and Moskowsky (1952) | 184 | 49·6 |
| Winder, Escher and Mantel (1966) | 112 | 49·53 |
| Dibbelt, Müller and Ehlers (1962) | 161 | 49·7 |
| Way (1954) | 153 | 51·5 |
| Kottmeier (1959) | 617 | 51·6 |
| Boutselis, Bair, Vorys and Ullery (1963) | 269 | 49 |
| Scheffey, Thudium and Farrell (1943) | 127 | 49 |
| Kimbell (1954) | 173 | 49·5 |
| Twombly and Jacobowitz (1962) | 82 | 48·4 |

United Manchester Hospitals (St. Mary's Hospitals). A control group of 300 women suffering from non-malignant disease was drawn from the general surgical and medical wards of the United Manchester Hospitals (Manchester Royal Infirmary); this control group was age matched in 5 year groups with the adenocarcinoma series. In selecting the control group any patient suffering from a condition known to be specifically associated with obesity, hypertension, diabetes mellitus or endocrine disorder was excluded. The composition of the control group is shown in Table III.

TABLE III.—*Composition of Control Series*

| Disease | Number of cases |
|---|---|
| Appendicitis . . . . . | 75 |
| Chronic peptic ulcer . . . . | 59 |
| Chronic rheumatic heart disease . . | 56 |
| Diverticulitis . . . . . . | 28 |
| Hernia . . . . . . . | 18 |
| Chronic bronchitis . . . . | 18 |
| Ulcerative colitis . . . . | 12 |
| Haemolytic anaemia . . . . | 5 |
| Sarcoidosis . . . . . | 3 |
| Asthma . . . . . . . | 2 |
| Idiopathic steatorrhoea . . . | 10 |
| Miscellaneous . . . . . | 6 |
| Crohn's disease . . . . . | 8 |

For the purposes of this study hypertension was defined as a persistent diastolic pressure of 100 mm. of mercury or above; a patient was considered to have severe hypertension if her diastolic pressure was persistently 110 mm. of mercury or above.

Very few of the patients had been weighed and it was therefore difficult to obtain objective criteria of obesity. In all cases, however, the records contained a comment as to whether the patient was " thin ", " of average weight ", " obese " or " grossly obese " and patients falling into the latter two classifications were considered as obese in this study. It was thought that this classification was, despite its obvious limitations, reasonably valid and that any faults introduced by its use would be equally applicable to both the groups studied.

In hardly any of the cases, in either group, had glucose tolerance tests been performed, though all patients had had their urine tested for glycosuria. A patient was therefore classed as diabetic if either she was known to be suffering from established diabetes mellitus before the onset of her current illness and was receiving treatment for this condition or if urine testing during her current illness showed glycosuria that was proven by blood studies to be due to diabetes mellitus.

### RESULTS

The age distribution of patients with endometrical adenocarcinoma is shown in Table IV and is similar to that found in other series.

TABLE IV.—*Age Distribution of Cases of Endometrial Adenocarcinoma*

| <40 | 41–45 | 46–50 | 51–55 | 56–60 | 61–65 | 66–70 | 71–75 | >75 |
|---|---|---|---|---|---|---|---|---|
| 5 | 12 | 31 | 68 | 67 | 47 | 35 | 25 | 10 |

*Age of menopause.* The mean age at which a natural menopause occurred in the adenocarcinoma group was 49·1 years (standard deviation = $\pm$ 4·7 years). The mean age of natural menopause in the control series was 47·9 years (standard deviation = 4·36 years). The standard error of the difference of the means is 0·55 and the observed difference of 1·2 years is therefore probably significant.

It has been suggested that it is probably more pertinent to consider the incidence of patients in each group whose menopause occurred after the age of 52 (Peel, 1956). In the adenocarcinoma series 53 patients had not reached their menopause by the age of 53, whilst in the control group 33 women were still premenopausal at this age. This difference is probably significant (chi square $= 4{\cdot}9$ $P =$ less than 0·05).

*Marital state.* In the adenocarcinoma series 58 patients (19·33%) were unmarried whilst in the control series 35 women (11·66%) were single. This difference is probably significant (chi square $= 6{\cdot}2$ $P =$ less than 0·05).

*Parity.* Amongst the patients with endometrial adenocarcinoma 112 (37·3%) were nulliparous as were 71 women (23·7%) in the control series. This is a significant difference (chi square $= 12{\cdot}58$ $P =$ less than 0·001). If the incidence of nulliparity is considered only in those women who were married then the figures for the adenocarcinoma and the control series respectively were 54 of 242 patients (22·3%) and 36 of 265 patients (13·6%). This difference is still probably significant (chi square $= 6{\cdot}017$ $P =$ less than 0·05).

*Hypertension.* One hundred and six (35·33%) of the patients with endometrial adenocarcinoma were hypersensitive as were 75 (25%) of the women in the control series. This difference in incidence is a significant one (chi square $= 7{\cdot}1$ $P = 0{\cdot}01$). Within these groups were 47 (15·66%) cases of severe hypertension in the adenocarcinoma series and 29 (9·66%) severely hypertensive women in the control series. This difference is also probably significant (chi square $= 4{\cdot}35$ $P =$ less than 0·05).

*Obesity.* In the adenocarcinoma group 102 patients (34%) were classed as obese as compared to 94 patients (31·33%) in the control series. This difference in incidence is clearly not significant.

*Diabetes mellitus.* Thcre were 11 diabetics (3·66%) amongst the patients with adenocarcinoma as compared to 9 diabetics (3%) in the control series; this is not a significant difference.

*Thyroid disease.* A history of past or present thyroid disease was noted in 13 (4·33%) of the patients with endometrical adenocarcinoma and in 18 (6%) of the women in the control group.

*Concomitant malignant disease.* Six of the patients with endometrial adenocarcinoma had a concomitant extragenital malignant tumour as did also 6 patients in the control series.

### DISCUSSION

In a study of this type it is necessary to consider the validity of the control group. The one that we have used was drawn from the same patient population as were the cases of endometrial adenocarcinoma: this is a necessary precaution in so far as it is probable that both the incidence of obesity and the average age of menopause are subject to a quite considerable regional and socio-economic variation. Although every effort was made to exclude from the control group any patient suffering from a disease known to be associated with hypertension, diabetes or endocrine disorder it is almost impossible to exclude totally conditions uninfluenced by obesity; thus it is probable that cases of diverticulitis, hernia and chronic bronchitis may be exacerbated by obesity and therefore more likely to be admitted to hospital. However, the reverse also applies and patients with ulcerative colitis, Crohn's disease or steatorrhoea are unlikely to be overweight; this second group probably neutralises the tendency to overestimate the incidence of obesity that it is virtually inherent in any control series that is drawn from a hospital population. It may be argued that it would be better to use a control series of well women; quite apart from the problems entailed in obtaining data from an age matched group of such women, it is extremely difficult to reduplicate the socio-economic stratification that is characteristic of any particular hospital.

If the validity of the control group is accepted then it is clear that endometrial adenocarcinoma is not, in the population studied, specifically associated with obesity. It may be thought that the incidence of obesity in the adenocarcinoma group had been underestimated in so far as patients with a malignant tumour often lose weight before their illness is diagnosed. All patients in this group had, however, been questioned as to weight loss and the number who had lost any appreciable amount of weight was too small to be of any real significance.

It is also apparent that there is, in our patient population, no excess of diabetics amongst patients with endometrial adenocarcinoma. The incidence of such patients in our series of 3·6% is similar to that found by Peel (1956), Miller *et al.* (1962), Javert and Renning (1963) and Wynder, Escher and Mantel (1966), but is totally at variance with those of other workers in whose series between 25% and 50% of patients with endometrial adenocarcinoma were classed as diabetic. It is quite clear that two totally different parameters are being cited in these various studies, one being the incidence of true diabetics and the other the incidence of disturbance in carbohydrate metabolism; this being so any attempt to compare results is a pointless exercise. There is, however, no convincing evidence that frank diabetes mellitus occurs unduly commonly in patients with endometrial adenocarcinoma and it may be argued that this is a more relevant finding than is the incidence of abnormal glucose tolerance tests in this condition. Firstly, very few studies have been made of the incidence of abnormal glucose tolerance tests in healthy women of the age group in which endometrial adenocarcinoma occurs; certainly, studies of glucose metabolism have not been made in age matched control groups from the same population pool in any reported series thus making it difficult to assess the significance or otherwise of any data obtained from the adenocarcinoma patients. Secondly, those studies in which there has been a high incidence of abnormal glucose tolerance tests in patients with endometrial adenocarcinoma have often been those in which these patients were also reported as having an unusually high incidence of obesity; as the relationship between obesity and disturbances in glucose metabolism is well known this factor may have considerably biased their results. Thirdly, it is possible, indeed probable, that malignant tumours of any type may have a non-specific effect on the glucose tolerance test (Vander, 1959). Lastly, there is the important consideration that exogenous oestrogens can impair glucose tolerance (Goldman and Ovadia, 1969): as there is considerable evidence to suggest that hyperoestrogenism may, in a proportion of cases, be an aetiological factor in endometrial adenocarcinoma (Andrews, 1961) it could be expected that such patients could also have abnormal glucose tolerance tests because of their excess endogenous oestrogen. All these factors complicate the study of glucose tolerance tests in endometrial adenocarcinoma and dilute the significance of any results obtained thus making it more profitable to study simply the incidence of diabetes mellitus.

Our study does confirm the association between endometrial adenocarcinoma and hypertension, this association holding for both mild and severe hypertension. It can be argued that the excess of hypertension often noted in patients with this tumour reflects the high incidence of associated obesity and is due to the misleading readings obtained by taking blood pressure measurements from a very plump arm. This does not, however, hold true for our series in which there was no excess of obese patients to the adenocarcinoma group.

Our results also confirm that patients with endometrial adenocarcinoma show a significant tendency to be nulliparous, to be unmarried and to have a late menopause; we have not been able to confirm any association with thyroid disease or with extragenital malignant neoplasm.

These findings require an interpretation and it could be suggested that they are, when taken together, explicable on the basis of an abnormality in adrenal function, this being responsible both for a disturbance in oestrogen metabolism and for the presence of hypertension. It is well established that tumours and marked hyperplasia of the adrenal glands can cause such abnormalities, *e.g.* in Cushing's syndrome, but it is as yet far from clear whether or not relatively minor adrenal lesions, such as mild cortical hyperplasia or small cortical adenomata, can also produce this complex. It has indeed been claimed that many cases of essential hypertension are due to relatively minor abnormalities of the adrenals (Conn, 1964) but the truth or otherwise of this contention is not yet fully established. The hypothesis of adrenal overactivity is an attractive one for it would explain not only the constitutional background of patients with endometrial adenocarcinoma but can also be incriminated as an aetiological factor in the development of this neoplasm, for which a basis of hyperoestogenism is, in a proportion of cases, well established (Andrews, 1961). This hypothesis is sufficiently feasible for us to suggest that a much more detailed study of adrenal function and structure in cases of endometrial adenocarcinoma would be a worthwhile undertaking.

### REFERENCES

ANDREWS, W. C.—(1961) *Obstetl gynec. Surv.*, **16**, 747.
BASTIAANSE, M. A. VAN B.—(1952) *J. Obstet. Gynaec. Br. Emp.*, **59**, 611.
BOUTSELIS, J. G., BAIR, J. R., VORYS, N. AND ULLERY, J. C.—(1963) *Am. J. Obstet. Gynec.*, **85**, 994.
CONN, J. W.—(1964) *J. Am. med. Ass.*, **190**, 222.
DAMON, A.—(1960) *J. natn. Cancer Inst.*, **24**, 483.
DAVIS, E. W.—(1964) *Am. J. Obstet. Gynec.*, **88**, 163.
DIBBELT, L., MÜLLER, H. G. AND EHLERS, F.—(1962) *Z. Geburtsh. Gynäk.*, **160**, 1.
DUNN, L. J., MERCHANT, J. A., BRADBURY, J. T. AND STONE, D. B.—(1968) *Archs intern. Med.*, **121**, 246.
GARNET, J. D.—(1966) in ' New Concepts in Gynecological Oncology ', edited by G. C. Llewis, W. B. Went and R. M. Jaffe. Philadelphia (F. A. Davis Co.).
GOLDMAN, J. A. AND OVADIA, J. L.—(1969) *Am. J. Obstet. Gynec.*, **103**, 172.
GRAHAM, J. B.—(1964) *Obstet. Gynec., N.Y.*, **23**, 176.
HOYNCK VAN PAPENDRECHT, H. P.—(1965) *Ned. Tijdschr. Geneesk.*, **109**, 68.
JAVERT, C. T. AND RENNING, E. L.—(1963) *Cancer, N.Y.*, **16**, 1057.
KIMBELL, C. W. A.—(1954) *Proc. R. Soc. Med.*, **47**, 895.
KOTTMEIER, H. L.—(1959) *Am. J. Obstet. Gynec.*, **78**, 1127.
LYNCH, H. T., KRUSH, A. J., LARSEN, A. L. AND MAGNUSON, C. W.—(1966) *Am. J. med. Sci.*, **252**, 381.
MILLER, M. C., ROBERTSON, G. T., SWANSON, W. C. AND WALKER, J.—(1962) *J. Obstet. Gynaec. Br. Commonw.*, **69**, 553.

MOSS, W. T.—(1947) *Am. J. Roentg.*, **58**, 203.
PALMER, J. P., REINHARD, M. C., SADUGOR, M. G. G. AND GOLTZ, H. L.—(1949) *Am. J. Obstet. Gynec.*, **58**, 457.
PEEL, J. H.—(1956) *Am. J. Obstet. Gynec.*, **71**, 718.
PENTECOST, M. P. AND BRACK, C. B.—(1959) *Sth. med. J., Nashville*, **52**, 190.
ROBERTS, D. W. T.—(1961) *J. Obstet. Gynaec. Br. Commonw.*, **68**, 132.
SCHEFFEY, L. C., THUDIUM, W. J. AND FARRELL, D. M.—(1943) *Am. J. Obstet. Gynec.*, **46**, 786.
SMITH, G. VAN S.—(1941) *New Engl. J. Med.*, **225**, 608.
SOMMERS, S. C. AND MEISSNER, W. A.—(1957) *Cancer, N.Y.*, **10**, 516.
THIEDE, H. A. AND LUND, C. J.—(1962) *Obstet. Gynec., N.Y.*, **20**, 149.
TWOMBLY, G. H. AND JACOBOWITZ, W. E.—(1962) *N.Y. St. J. Med.*, **62**, 2194.
VANDER, J. B.—(1959) *Am. J. Obstet. Gynec.*, **77**, 243.
WATERMAN, G. W., RAPHAEL, S. I. AND MOSKOSKY, W.—(1952) *Am. J. Obstet. Gynec.*, **64**, 1073.
WAY, S.—(1954) *J. Obstet. Gynaec. Br. Emp.*, **61**, 46.
WYNDER, E. L., ESCHER, C. C. AND MANTEL, N.—(1966) *Cancer, N.Y.*, **19**, 489.
YAHIA, C., BENIRSCHKE, K. AND STURGIS, H. S.—(1963) in ‘ Progress in Gynaecology ’, Vol. 4. London (Heinemann).